# Comment on: Pettersson *et al*. Qdenga-induced dengue caused by minor DENV-2 subvariant(s) in the vaccine, with two amino acid substitutions in the E protein

**DOI:** 10.1093/jtm/taaf127

**Published:** 2026-01-13

**Authors:** Vianney Tricou, Jill Livengood, Mayuri Sharma, Olaf Zent, Martina Rauscher, Yin Xiang Setoh, Carolina Brünner, Shibadas Biswal, Walid Kandeil

**Affiliations:** Vaccine Business Unit, Takeda Pharmaceuticals International AG, Thurgauerstrasse 130, 8152 Glattpark-Opfikon (Zurich), Switzerland; Vaccine Business Unit, Takeda Vaccines Inc., 75 Sidney St, Cambridge, MA 02139, USA; Vaccine Business Unit, Takeda Vaccines Inc., 75 Sidney St, Cambridge, MA 02139, USA; Vaccine Business Unit, Takeda Pharmaceuticals International AG, Thurgauerstrasse 130, 8152 Glattpark-Opfikon (Zurich), Switzerland; Vaccine Business Unit, Takeda Pharmaceuticals International AG, Thurgauerstrasse 130, 8152 Glattpark-Opfikon (Zurich), Switzerland; Growth & Emerging Markets, Takeda Pharmaceuticals International AG Singapore Branch, 8 Marina Boulevard, #15-01 Marina Bay Financial Centre Tower 1, 018981 Singapore; Takeda Pharma A/S, Delta Park 45, 2665 Vallensbæk Strand, Denmark; Vaccine Business Unit, Takeda Vaccines Inc., 75 Sidney St, Cambridge, MA 02139, USA; Vaccine Business Unit, Takeda Pharmaceuticals International AG, Thurgauerstrasse 130, 8152 Glattpark-Opfikon (Zurich), Switzerland

**Keywords:** vaccine, dengue, TAK-003

Dear Editor,

We write in response to a publication from Pettersson *et al*. (Qdenga-induced dengue caused by minor DENV-2 subvariant(s) in the vaccine, with two amino acid substitutions in the E protein).^1^ TAK-003 (Qdenga®) is a live-attenuated tetravalent dengue vaccine based on a dengue virus (DENV)-2 backbone and includes viral constructs with pre-membrane (prM) and envelope protein regions of DENV-1, DENV-3 and DENV-4.^2^ It is approved in >40 countries and available in >25 countries. The authors describe dengue-like symptoms in a TAK-003 recipient, along with a minor DENV-2 subvariant. We would like to provide context for and clarify the results.

In the report, the traveller experienced mild symptoms (fever, headache, malaise, arthralgia, myalgia and rash), not requiring hospitalization, and fully recovered within 2 days. These manifestations, 16 days post-vaccination, are consistent with vaccine viraemia-induced symptoms.^3^ Transient viraemia of predominantly DENV-2 and mild/moderate symptoms post-vaccination have also been observed in our trials,^3^ and this is reflected in the product label.^4^ These observations do not represent any new/previously unreported adverse events, nor do they suggest a confirmed case of dengue fever caused by TAK-003. An analysis of pooled safety data from several phase 1 and 2 studies showed that vaccine viraemia was associated with systemic symptoms that likewise occurred in participants without viraemia, underscoring the non-specific nature of these symptoms.^3^ Additionally, TAK-003 has been evaluated in children with prior Japanese encephalitis or yellow fever (YF) vaccination, demonstrating an acceptable safety profile, with no clinically relevant impact.^5^ Therefore, the traveller’s prior vaccination against YF raises no concern.

The authors reported amino acid substitutions in the proteome of TAK-003. Except for the two mutations in the envelope gene, the other minor variations have been previously documented or published.^2^ The prM-11 change likely reflects a data source discrepancy; the patient amino acid matches our master virus seed. prM-7 also matches the reference strain. Viral quasi-species sometimes occur in live-attenuated RNA vaccines and, importantly, the main attenuation markers in the backbone of TAK-003 remained intact. Genetic stability of these markers, particularly NS1-53 and NS3-250, has been thoroughly investigated,^2^ and they have been shown to be associated with low levels of viraemia,^3^ which may help restrain quasi-species diversity. This makes reversion to virulence due to the reported substitutions unlikely. Additionally, routine quality tests on bulk drug substance lots require confirmation of key attenuating mutations before incorporation into the drug product, as these are essential for safety. The mere presence of a subvariant does not equate to causation of dengue fever.

In conclusion, no new/unusual adverse events in relation to TAK-003 administration were reported by Pettersson *et al*. Our interpretation of the results is that the reported symptoms are more consistent with those associated with vaccine viraemia. Takeda continues to monitor safety and report findings to regulators and national surveillance systems. As of September 2025, ~ 19 million TAK-003 doses have been distributed. The overall benefit–risk profile remains favourable.
